# Operation of trans-thylakoid thiol-metabolizing pathways in photosynthesis

**DOI:** 10.3389/fpls.2013.00476

**Published:** 2013-11-27

**Authors:** Mohamed Karamoko, Stéphane T. Gabilly, Patrice P. Hamel

**Affiliations:** ^1^Department of Molecular Genetics, The Ohio State UniversityColumbus, OH, USA; ^2^Department of Molecular and Cellular Biochemistry, The Ohio State UniversityColumbus, OH, USA

**Keywords:** thylakoid lumen, photosynthesis, disulfide, thioredoxin

## Abstract

Thiol oxidation to disulfides and the reverse reaction, i.e., disulfide reduction to free thiols, are under the control of catalysts *in vivo*. Enzymatically assisted thiol-disulfide chemistry is required for the biogenesis of all energy-transducing membrane systems. However, until recently, this had only been demonstrated for the bacterial plasma membrane. Long considered to be vacant, the thylakoid lumen has now moved to the forefront of photosynthesis research with the realization that its proteome is far more complicated than initially anticipated. Several lumenal proteins are known to be disulfide bonded in *Arabidopsis*, highlighting the importance of sulfhydryl oxidation in the thylakoid lumen. While disulfide reduction in the plastid stroma is known to activate several enzymatic activities, it appears that it is the reverse reaction, i.e., thiol oxidation that is required for the activity of several lumen-resident proteins. This paradigm for redox regulation in the thylakoid lumen has opened a new frontier for research in the field of photosynthesis. Of particular significance in this context is the discovery of trans-thylakoid redox pathways controlling disulfide bond formation and reduction, which are required for photosynthesis.

Thiol-disulfide chemistry refers to the oxidation of thiols (sulfhydryls) into disulfides and also the reversible reaction, i.e., the reduction of the disulfides to free thiols. *In vivo*, both chemical reactions are catalyzed by dedicated enzymes in cellular compartments where disulfide bonds and reduced thiols in protein targets need to be maintained at all times. Thiol-disulfide chemistry has been best studied in bacteria where it is required for the biogenesis of the periplasmic compartment (Denoncin and Collet, 2013). It has now become apparent that catalyzed thiol-disulfide reactions also operate in the mitochondrial intermembrane space (IMS) and the thylakoid lumen, which are topologically equivalent to the bacterial periplasm. In mitochondria, the Mia40/Erv1 proteins were discovered to be key enzymes of a disulfide relay system driving the import of cysteine-rich proteins into the IMS by an oxidative folding mechanism (Herrmann and Riemer, 2012). The thylakoid lumen has long been viewed as a vacant compartment, with the exception of the oxygen evolving complex (OEC) and some of the redox carriers (e.g., cytochrome *f*, Rieske, and plastocyanin-or its substitute cytochrome *c*_6_) involved in electron transfer during the light reactions of photosynthesis. This dogma has now been revised with the realization that numerous molecules reside in the thylakoid lumen in addition to the previously known photosynthetic proteins. Proteomics studies revealed that the lumenal proteome (~80–200 proteins) is far more complicated than initially anticipated and includes proteases, chaperones, isomerases, redox enzymes, and other proteins of unknown activities (Kieselbach and Schröder, 2003). It is possible that the proteins revealed by proteomics regulate photosynthesis or other yet-to-be discovered processes unrelated to photosynthesis. A recent study demonstrated that lumenal PPD5 is not involved in photosynthetic electron transfer reactions, but rather controls the synthesis of strigonolactone, a plant hormone regulating axillary bud formation (Roose et al., 2011). There is mounting evidence that several lumenal proteins, including components of the photosynthetic chain, contain one or several disulfide bonds (Kieselbach, 2013; **Table 1**). However the functional importance of the disulfide(s) has only been examined for a small number of these proteins. The molecular mass of disulfide bonded proteins in the thylakoid lumen range from 10 to 55 kDa and the disulfide bond forming cysteines do not appear to occur in a motif. This is at contrast with most of the disulfide bonded molecules in the mitochondrial IMS which are small proteins (6–18 kDa) containing two disulfide bonds in either a C(*X*)_3_C or a C(*X*)_9_C motif (Sideris and Tokatlidis, 2010). Interestingly, while disulfide bond reduction in stroma-localized targets serves to activate several enzymes (Meyer et al., 2009), it appears that it is the reverse reaction, i.e., disulfide bond formation, that is required for the activities of several lumen-resident proteins (Buchanan and Luan, 2005).

**Table 1:** Lumenal disulfide bonded proteins in *Arabidopsis*.

protein	Gene locus	Classification	Function
PsbO1 (2 cys, **1 SS**)	AT5G66570	PSII subunit	Oxygen evolution
PsbO2 (2 cys, **1 SS**)****	AT3G50820	PSII subunit****	Oxygen evolution
PSI-N (4 cys, **2 SS**)	AT5G64040	PSI subunit	Regulation of PSI activity?
Rieske (2 cys, **1 SS**)	AT4G03280	Cytochrome *b*_6_*f* subunit	Electron transfer in *b*_6_*f* complex
Cyt *c*_6A_ (2 cys, **1 SS**)	AT5G45040**	*c*-type cytochrome	Unknown
STT7/STN7 (2 cys, 1 SS)	AT1G68830	Kinase	State transition
FKBP13 (4 cys, **2 SS**)	AT5G45680	Peptidyl-prolyl *cis*-*trans* isomerase	Unknown
FKBP20-2 (2 cys, **1 SS**)	AT3G60370	Peptidyl-prolyl *cis*-*trans* isomerase	PSII supercomplex assembly
PrxQ (2 cys, **1 SS**)	AT3G26060	Peroxiredoxin	Unknown
VDE (13 cys, ≥**4 SS**)	AT1G08550	Violaxanthin de-epoxidase	Photoprotection
PPD6 (2 cys, **1SS**)	AT3G56650	PsbP domain protein	Unknown
TL15 (2 cys, 1 SS)	AT2G44920	Pentapeptide repeat	Unknown
TL17 (4 cys, **2 SS**)	AT5G53490	Pentapeptide repeat	Unknown
TL20.3 (4 cys, ≥1 SS)	AT1G12250	Pentapeptide repeat	Unknown
TL29 (2 cys, 1 SS)	AT4G09010	Ascorbate peroxidase	Unknown
CtD1 (5 cys, ≥1 SS)	AT4G17740	Protease	Processing of D1 subunit
CtD1-like (4 cys, ≥1 SS)	AT5G46390	Protease	Unknown
Deg5 (2 cys, 1 SS)	AT4G18370	Protease	Degradation of lumenal proteins?

*The number of lumenal cysteines (cys) that can form an intramolecular disulfide and the number of disulfides (SS) are indicated in parenthesis. The number of disulfide is experimentally determined. Bolded font indicates that the presence and the number of the disulfide has been confirmed. “≥*n* SS” indicates that the protein contain at least n disulfide(s). Experimental evidence for the presence of disulfides comes from a combination of thiol trapping/labeling experiments, structure determination or the finding that the enzymatic activity is sensitive to disulfide reducing agents such as DTT or abolished by site-directed mutagenesis of conserved cysteine residues. Several single cysteine containing lumenal proteins (not indicated here) were recovered in thiol trapping experiments, suggesting that intermolecular disulfides can be formed. See references (Hall et al., 2010; Hall, 2012; Kieselbach, 2013).*

In bacteria, the requirement for disulfide bond reduction in the periplasmic space was established mainly through studies of cytochrome *c* maturation. Cytochromes *c* are metalloproteins with one or several hemes covalently linked to a C*XX*CH motif on the protein (Thony-Meyer, 1997; Hamel et al., 2009). The need for a disulfide-reducing activity in the context of bacterial cytochrome *c* assembly seemed obvious because the periplasmic space is also the compartment where cysteine-containing proteins are oxidized by dedicated catalysts (Denoncin and Collet, 2013). The current view is that the C*XX*CH motif is oxidized after translocation of apocytochromes *c* to the periplasmic space and then reduced by a reducing pathway to provide free sulfhydryls for ligation with heme (Bonnard et al., 2010; Sanders et al., 2010). This question had received little attention in the context of plastid cytochromes *c* because thiol-disulfide chemistry as a catalyzed process was not believed to take place in the thylakoid lumen, the compartment where heme attachment to apocytochromes *c* occurs. The discovery of trans-thylakoid redox pathways controlling disulfide bond formation and reduction in *Chlamydomonas* and *Arabidopsis* has now changed this perception.

## TWO DISULFIDE-REDUCING PATHWAYS OPERATE IN THE LUMEN

**FIGURE 1:**
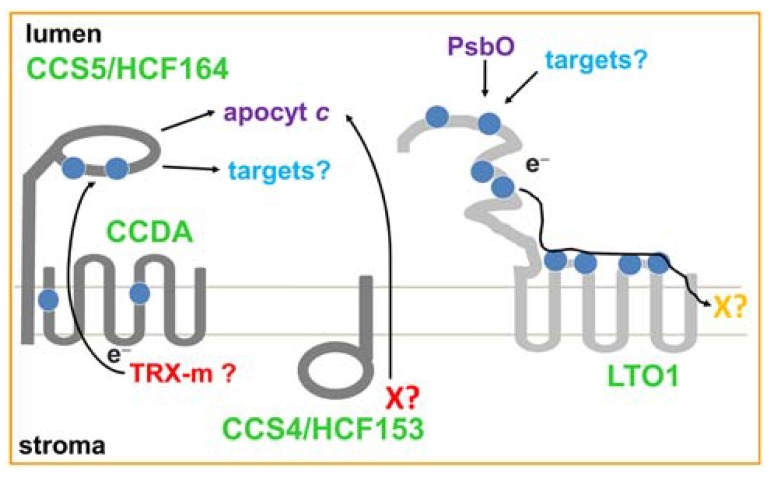
**FIGURE 1. *Trans*-thylakoid thiol metabolizing pathways.** The disulfide-reducing (CCDA, CCS5/HCF164 and CCS4/HCF153) and thiol-oxidizing pathways (LTO1) are represented in dark and light grey, respectively. Cysteines are represented as blue balls. Electron (*e*^-^) routes are indicated by arrows. The topology of CCS4/HCF164 is hypothetical but the positive-inside rule predicts a stromal localization for the C-terminal domain of the protein. CCS5/HCF164 is a membrane anchored lumen-facing thioredoxin-like protein. LTO1 contains a VKOR-like membrane domain and a lumen-facing thioredoxin-like domain. Stromal reductants for the disulfide reducing pathways are indicated in red. The electron acceptor for the thiol-oxidizing pathway is indicated in orange. Thioredoxin-*m* (Trx-m) is the possible reductant for the CCDA-CCS5/HCF164 pathway. The CCS4/HCF153 pathway might be involved in the transport of a reductant such as glutathione or ascorbate (X?). The final electron acceptor of the LTO1-dependent pathway is not known and is postulated to be a phylloquinone (X?). CCDA and LTO1 topology was deduced using PhoA/LacZ topological reporters (Page et al., 2004; Feng et al., 2011; Karamoko et al., 2011). The thylakoid membrane is shown in light grey.

A central component of the bacterial disulfide-reducing pathways is the thiol-disulfide oxido-reductase of the DsbD family (Cho and Collet, 2013). In bacteria, members of this family (CcdA, DsbD, ScsB) are cytoplasmic membrane proteins conveying reducing power from the cytosol to the active sites of several target molecules in the periplasm. Reducing power is transferred across the cytoplasmic membrane through sequential thiol-disulfide exchanges (Cho and Collet, 2013). One target required for cytochrome *c* assembly is the membrane-bound, periplasm-facing, thioredoxin-like protein (CcmG/ResA/CcsX). CcmG/ResA/CcsX is postulated to reduce a disulfide in the C*XX*CH heme-binding site of apocytochrome *c* prior to heme ligation to the cysteines (Bonnard et al., 2010; Sanders et al., 2010). In the plastid lumen, the involvement of a disulfide-reducing pathway was first suspected based on the presence of CCDA, an ortholog of bacterial CcdA from the DsbD family, at the thylakoid membrane (Page et al., 2004; Motohashi and Hisabori, 2010; **Figure 1**). Another component in the plastid is HCF164 (High Chlorophyll Fluorescence), a membrane-anchored, lumen-facing, thioredoxin-like protein with similarity to bacterial CcmG/ResA/CcsX (Lennartz et al., 2001; **Figure 1**). The disulfide-reducing activity of HCF164 was inferred from the fact that a recombinant form of its lumenal domain displays disulfide reductase activity *in vitro* (Lennartz et al., 2001; Motohashi and Hisabori, 2006). Loss of function of CCDA or HCF164 in *Arabidopsis* produces a photosynthetic-deficient phenotype due to a defect in cytochrome *b*_6_*f* complex assembly (Lennartz et al., 2001; Page et al., 2004). However, the biochemical activity and site of action of CCDA and HCF164 in the assembly process remained unknown until the characterization of the *Chlamydomonas ccs4* and *ccs5* mutants (Xie et al., 1998). The *ccs4* and *ccs5* mutants (*ccs* for *c*ytochrome *c s*ynthesis), which are partially photosynthetic deficient, display a block in the conversion of apo to holoform of lumen resident cytochromes *c*, namely membrane-bound cytochrome *f* and soluble cytochrome *c*_6_. The *ccs* mutants are deficient for cytochrome *b*_6_*f* assembly, which is dependent on cytochrome *f*, the thylakoid membrane-bound cytochrome *c*. This assembly defect is at the step of heme attachment to apocytochromes *c*, a chemical reaction taking place in the thylakoid lumen (Howe and Merchant, 1992, 1993, 1994; Xie et al., 1998). The *Chlamydomonas CCS5* gene was cloned and shown to encode a thioredoxin-like protein with similarity to *Arabidopsis* HCF164 (Gabilly et al., 2010; **Figure 1**). *Arabidopsis* HCF164 can complement the cytochrome *c* assembly defect when expressed from the plastid genome of the *Chlamydomonas ccs5* mutant, demonstrating that HCF164 and CCS5 are functionally equivalent. This also suggests that the defect in cytochrome *b*_6_*f* assembly in the *Arabidopsis hcf164* mutant is caused by a block in the conversion of apo to holocytochrome *f*. The role for CCS5 as an apocytochrome *c* disulfide reductase in cytochrome *c* assembly was formulated based on the following findings: (a) the *ccs5*-null mutant can be chemically rescued *in vivo* by application of exogenous thiols (such as DTT); (b) CCS5 interacts with plastid apocytochromes *c* in yeast two-hybrid assays; and c) a recombinant form of CCS5 is able to reduce a disulfide bonded C*XX*CH in apocytochrome *c in vitro* (Gabilly et al., 2010). The proposed model is that the thiol-disulfide oxido-reductase CCDA and the thioredoxin-like protein CCS5/HCF164 define a trans-thylakoid pathway for the delivery of reductants from the stroma to the lumen (Lennartz et al., 2001; Page et al., 2004; Motohashi and Hisabori, 2006; Gabilly et al., 2010; Motohashi and Hisabori, 2010; Gabilly et al., 2011). This pathway is required for cytochrome *b*_6_*f* assembly but it is conceivable that it also acts as the transducer of reducing power to regulate the thiol-dependent activity of other lumenal targets (Motohashi and Hisabori, 2006). For instance, *in vitro* and *in organello* experiments showed that the reduction of the two disulfides in PSI-N (**Table 1**), a subunit of photosystem I (PSI), is dependent upon HCF164. The source of reducing power on the stromal side is not known but *in organello* experiments support the role of the thioredoxin Trx-*m* as a possible electron donor to CCDA and HCF164 (Motohashi and Hisabori, 2010).

The *Chlamydomonas CCS4* gene encodes a small protein with limited similarity to *Arabidopsis* HCF153, a thylakoid membrane anchored protein required for the assembly of the cytochrome *b*_6_*f* complex (Lennartz et al., 2006; Gabilly et al., 2011; **Figure 1**). The CCS4 protein contains an N-terminal hydrophobic stretch that could serve as a membrane anchor and a C-terminal domain rich in charged residues. On the basis of the positive-inside rule that governs the topology of bacterial and thylakoid membrane proteins (von Heijne, 1989; Gavel et al., 1991), the C-terminal domain of CCS4 is predicted to be exposed to the stromal side of the thylakoid membrane. Surprisingly, CCS4 does not display any motif or residue (such as cysteines) suggesting a role in thiol-based redox chemistry. Yet, the thiol-dependent photosynthetic rescue of the *ccs4* mutant and the suppression of the *ccs4* phenotype by ectopic expression of CCDA, the thiol/disulfide oxido-reductase of the DsbD family, at the thylakoid membrane, confirms the activity of CCS4 in a disulfide-reducing pathway for cytochrome *c* assembly. Moreover, the CCDA-dependent suppression of the *ccs4* mutant confirms the placement role of CCDA in plastid cytochrome *c* maturation. Indeed, earlier studies in *Arabidopsis* supported, but did not establish, the requirement of plastid CCDA in the conversion of apo- to holocytochromes *c* (Page et al., 1997; Motohashi and Hisabori, 2010). Interestingly, the *ccs4-*null *ccs5-*null double mutant displays a synthetic phenotype characterized by a complete loss of *b*_6_*f* assembly, an indication that CCS4 and CCS5 are redundant. This functional redundancy suggests that CCS4 might control a different disulfide-reducing pathway than the CCS5/HCF164 dependent one (**Figure 1**). The CCDA-dependent suppression of the *ccs4* mutant can be explained by a compensatory effect due to enhanced expression of the thiol-disulfide oxido-reductase CCDA. At the present time, the activity of CCS4 in such a pathway remains elusive. While the CCDA dependent pathway relies on the transfer of reductants through sequential thiol-disulfide exchanges, it is conceivable that CCS4 controls the transport across the thylakoid membrane of a molecule acting as a reducing agent (**Figure 1**). In bacteria, operation of such redundant routes for export of reductant to the periplasm have been postulated (Pittman et al., 2005). The nature of the exported reductant can only be speculated upon but glutathione or ascorbate is an obvious candidate. While the presence of glutathione in the thylakoid lumen remains to be established, ascorbate is known to function in this compartment as an alternative electron donor to PSII and a co-factor for violaxanthin de-epoxidase (VDE) dependent photoprotection (Tóth et al., 2013).

## DISCOVERY OF A DISULFIDE-FORMING ENZYME IN THE THYLAKOID LUMEN

In the periplasmic space of most proteobacteria, the thiol-oxidizing pathway consists of a disulfide bond catalyzing system defined by soluble DsbA and membrane-bound DsbB (Denoncin and Collet, 2013). DsbA catalyzes disulfide bridge formation in cysteine-containing substrates that are translocated across the membrane into the periplasmic space. DsbB operates by recycling reduced DsbA to its oxidized form with transfer of electrons to quinones, which are membrane-soluble redox carriers in the respiratory chain. The fact that no DsbA or DsbB-like enzymes can be detected in the genomes of photosynthetic eukaryotes or Cyanobacteria, the presumed ancestors of the chloroplast, reinforced the view that disulfide bond formation did not take place in the thylakoid lumen. However, the operation of catalyzed disulfide bond formation in the lumen is supported by the finding that bacterial alkaline phosphatase (PhoA), an enzyme requiring two disulfide bonds for activity, and basic pancreatic trypsin inhibitor BPTI (aprotinin), a molecule containing three disulfide bonds, are active when targeted to this compartment in tobacco (Sone et al., 1997; Bally et al., 2008; Tissot et al., 2008). A novel class of disulfide-forming enzymes with similarity to VKOR (Vitamin K epoxide Oxidoreductase) was recently recognized in some bacterial phyla lacking the typical DsbAB components (including Cyanobacteria) and in all photosynthetic eukaryotes (Dutton et al., 2008; Singh et al., 2008; Grossman et al., 2010). VKOR is well studied for its involvement in the reduction of vitamin K, a phylloquinone required as a co-factor for the γ-carboxylation of clotting factors in blood (Tie and Stafford, 2008). Recent work shows that the enzymatic activity of VKOR is also linked to oxidative folding of proteins in the ER lumen (Rishavy et al., 2011; Schulman et al., 2011). A first indication that the thylakoid lumen houses a thiol-oxidizing pathway came from the identification of a VKOR-like protein in Cyanobacteria (Singh et al., 2008). *In vitro* reconstitution of disulfide bond formation with the purified cyanobacterial enzyme demonstrated the sulfhydryl oxidase activity of the protein (Li et al., 2010). However, the localization of the protein at the thylakoid membrane and its relevant targets of action in the lumen were not documented (Singh et al., 2008). The identity of the thiol-oxidizing catalyst in the thylakoid lumen of plastids has now emerged through the discovery of LTO1 (Lumen Thiol Oxidoreductase 1), a thylakoid membrane protein containing a VKOR domain fused to a thioredoxin-like moiety (**Figure 1**; Furt et al., 2010; Feng et al., 2011; Karamoko et al., 2011). Topological studies using bacterial topological reporters established a lumenal location for the LTO1 domains carrying the redox motifs and conserved cysteines (**Figure 1**). Previous studies with bacterial VKOR-like proteins have demonstrated that the thioredoxin-like domain carries a DsbA-like activity while the VKOR-like central domain is functionally equivalent to DsbB (Singh et al., 2008; Dutton et al., 2010; Wang et al., 2011). In *Arabidopsis*, loss of LTO1 function is associated with a severe phototrophic growth defect (Karamoko et al., 2011; Lu et al., 2013). Measurements of the photosynthetic activity indicate that *lto1* mutants display a limitation in the electron flow from Photosystem II (PSII). In accord with these measurements, *lto1* mutants show a severe depletion of several of the structural subunits of PSII (including subunits of the OEC) but no change in the accumulation of the cytochrome *b*_6_*f* complex or PSI and no defect in the activity of ATP synthase. In a yeast two-hybrid assay, the lumen-facing thioredoxin-like domain of LTO1 was shown to interact with PsbO, a lumenal PSII subunit in the OEC known to be disulfide bonded (**Table 1**). *In vitro*, the thioredoxin-like domain of LTO1 is able to introduce a disulfide bond in the PsbO target when recombinant forms of the molecules are used. Because the redox state of the sulfhydryls in PsbO was shown to be a determinant for the stability of this subunit and also for PSII accumulation (Burnap et al., 1994; Nikitina et al., 2008; Hall et al., 2010), it is likely that loss of disulfide bond formation in PsbO in the *lto1* mutants accounts for the PSII assembly defect. It is not known if the ability of LTO1 to form a disulfide bond in PsbO is linked to the import of this protein into the lumen, similarly to the Mia40-dependent pathway in mitochondria (Herrmann and Riemer, 2012). *In organello* import experiments showed that PsbO translocates at a different site than PsbP, another subunit of the PSII OEC (Hashimoto et al., 1997). It is plausible that this translocation step is assisted by LTO1 but this awaits experimental testing.

The final electron acceptor of the LTO1-dependent disulfide bond forming pathway is currently unknown (**Figure 1**). It is likely to be a phylloquinone based on the fact that the *Arabidopsis* protein reduces phylloquinone in an *in vitro* enzymatic assay (Furt et al., 2010). The role of phylloquinone as a structural cofactor tightly bound to PSI is well documented (Brettel, 1997). However, the occurrence of a pool of phylloquinone that is not bound to PSI suggests phylloquinone might participate in redox processes in addition to the known electron transfer reactions through PSI (Gross et al., 2006; Lohmann et al., 2006). This pool may act as an electron acceptor for the LTO1-dependent disulfide bond forming pathway *in vivo*.

## OUTLOOK

It is conceivable that catalyzed thiol-oxidation in the lumen extends to other disulfide-bond containing targets in addition to PsbO (**Table 1**). *In vitro* experiments suggest that the thioredoxin-like domain of LTO1 is also able to catalyze the formation of the two disulfide bonds in FKBP13 (Lu et al., 2013), a peptidyl-prolyl *cis*-*trans* isomerase whose activity is dependent upon sulfhydryl oxidation (Gopalan et al., 2004; **Table 1**). It is not known if additional enzymes with sulfhydryl oxidase activity besides LTO1 also operate in the thylakoid lumen. In bacteria, the disulfide-reducing pathway is also required to maintain the reduction state of periplasmic oxido-reductases that shuffle disulfide bonds that are incorrectly formed (Kadokura and Beckwith, 2010; Depuydt et al., 2011). The need for disulfide bond isomerization is critical for proteins containing more than two cysteines such as VDE, an enzyme involved in photoprotection whose activity depends on sulfhydryl oxidation (Kanervo et al., 2005; **Table 1**). Interestingly, the activity of recombinant VDE was initially reported to be low, presumably because of improper protein folding due to incorrect disulfide linkages (Bugos and Yamamoto, 1996; Hieber et al., 2002). Expression in the cytosol of a bacterial strain engineered for disulfide bond formation and isomerization resulted in a high level of VDE activity for the purified enzyme, an indication that isomerization of disulfide bond in the lumen is also likely to be required for yielding an active enzyme (Saga et al., 2010). It is possible that LTO1 exhibits this activity based on the finding that its thioredoxin-like domain is active as a disulfide-bond isomerase *in vitro* (Lu et al., 2013). Another thylakoid membrane protein displaying disulfide-bond isomerase activity *in vitro* is LQY1, an enzyme required for the repair and re-assembly of photodamaged PSII (Lu, 2011). However, it is not known if the active site of LQY1 faces the lumenal or stromal side of the thylakoid membrane. Further experimental work is required to establish the molecular identity of the missing redox components defining the thiol-metabolizing pathways, identify their relevant targets of action and understand how they control photosynthesis or other processes in the thylakoid lumen.

## Conflict of Interest Statement

The authors declare that the research was conducted in the absence of any commercial or financial relationships that could be construed as a potential conflict of interest.
